# Effect of the Gene *doublesex* of *Anastrepha* on the Somatic Sexual Development of *Drosophila*

**DOI:** 10.1371/journal.pone.0005141

**Published:** 2009-04-02

**Authors:** Mercedes Alvarez, María Fernanda Ruiz, Lucas Sánchez

**Affiliations:** Centro de Investigaciones Biológicas (C.S.I.C), Madrid, Spain; Ecole Normale Supérieure de Lyon, France

## Abstract

**Background:**

The gene *doublesex (dsx)* is at the bottom of the sex determination genetic cascade and is transcribed in both sexes, but gives rise to two different proteins, DsxF and DsxM, which impose female and male sexual development respectively via the sex-specific regulation of the so-called sexual cyto-differentiation genes. The present manuscript addressed the question about the functional conservation of the tephritid *Anastrepha* DsxF and DsxM proteins to direct the sexual development in *Drosophila* (Drosophilidae).

**Methodology:**

To express these proteins in *Drosophila*, the GAL4-UAS system was used. The effect of these proteins was monitored in the sexually dimorphic regions of the fly: the foreleg basitarsus, the 5th, 6th and 7th tergites, and the external terminalia. In addition, we analysed the effect of *Anastrepha* DsxF and DsxM proteins on the regulation of *Drosophila yolk protein* genes, which are expressed in the fat body of adult females under the control of *dsx*.

**Conclusions:**

The *Anastrepha* DsxF and DsxM proteins transformed *doublesex* intersexual *Drosophila* flies into females and males respectively, though this transformation was incomplete and the extent of their influence varied in the different sexually dimorphic regions of the adult fly. The *Anastrepha* DsxF and DsxM proteins also behaved as activators and repressors, respectively, of the *Drosophila yolk protein* genes, as do the DsxF and DsxM proteins of *Drosophila* itself. Finally, the *Anastrepha* DsxF and DsxM proteins were found to counteract the functions of *Drosophila* DsxM and DsxF respectively, reflecting the normal behaviour of the latter proteins towards one another. Collectively, these results indicate that the *Anastrepha* DsxF and DsxM proteins show conserved female and male sex-determination function respectively in *Drosophila*, though it appears that they cannot fully substitute the latter's own Dsx proteins. This incomplete function might be partly due to a reduced capacity of the *Anastrepha* Dsx proteins to completely control the *Drosophila* sexual cyto-differentiation genes, a consequence of the accumulation of divergence between these species resulting in the formation of different co-adapted complexes between the Dsx proteins and their target genes.

## Introduction

Sex determination is the commitment of an embryo to either the male or female developmental pathway. A plethora of sex determination mechanisms exists; all of which are represented in insects [1], [2], [3]. In *Drosophila melanogaster*, the sex determination mechanism has been thoroughly analysed. The epistatic relationships between the sex determination genes in this species show that hierarchical interaction occurs among them (reviewed in [4]). The characterisation of these genes has shown that their control during development is governed by the sex-specific splicing of their products. The product of one gene controls the sex-specific splicing of the pre-mRNA from the downstream gene in the genetic cascade. *Sex-lethal (Sxl)* is at the top of this cascade; its product controls the splicing of its own pre-mRNA as well as the splicing of the pre-mRNA from the downstream gene *transformer (tra)*. The Tra product and the product of the constitutive gene *transformer-2 (tra-2)* control the sex-specific splicing of pre-mRNA from the gene *doublesex (dsx)*, which is transcribed in both sexes but gives rise to two different proteins, DsxF and DsxM. These are transcription factors that impose female and male sexual development respectively via the sex-specific regulation of the so-called sexual cytodifferentiation genes.

The gene *dsx* has been characterised in the dipterans *Megaselia scalaris* [5], [6], *Musca domestica* [7], *Anopheles gambiae* [8], in the fruit flies *Bactrocera tryoni* [9], *Bactrocera oleae* [10], *Bactrocera dorsalis* [11], *Ceratitis capitata* [12] and in twelve *Anastrepha* species [13], [14], in the lepidopteron *Bombyx mori* [15], [16] and in the hymenopteran *Apis mellifera* [17]. In all these species, *dsx* codes for male- and female-specific RNAs, which encode the male-specific and female-specific Dsx proteins.

The gene *dsx* of *Anastrepha* species is transcribed during development and in adult life in both sexes, but its primary transcript undergoes sex-specific splicing so that a different mRNA is produced in each sex. These mRNAs encode the female DsxF and male DsxM proteins; these have the amino-terminal region in common but differ in the carboxyl-terminal region. The comparison of *Aodsx* mRNA molecular organisation in males and females suggest that, in *Anastrepha*, the male-splicing pathway represents the default mode. The conceptual translation of the male and female *Anastrepha dsx* mRNAs shows that they encode two polypeptides of 397 and 319 amino acids respectively. Their comparison with the Dsx proteins of other insects shows that the degree of similarity is higher for the female-specific than for the non-sex specific and the male-specific regions. Particularly conserved are the OD1 and OD2 domains, which endow the Dsx proteins with the capacity to interact with other proteins and with DNA [18], [19].

Molecular evolutionary analysis (both at the nucleotide and amino acid levels) of *dsx* in different insects revealed a topology in good agreement with their owners' taxonomic relationships. The great majority of the nucleotide changes detected in the *dsx* gene of the analysed species were significantly synonymous, evidence that strong purifying selection has acted on *dsx* so that the functional structure of the Dsx proteins is preserved. Yet, the common region of DsxF and DsxM proteins appeared to be the main target for selection acting upon the long-term evolution of gene *dsx*. Although in a lesser extent if compared with common regions, the sex-specific segments of DsxF and DsxM proteins are also subject to purifying selection, as expected since they endow these proteins with a different, oppose transcriptional role that would be preserved across species [14].

*Anastrepha obliqua dsxF*-cDNA and *dsxM*-cDNA encoding the putative full-length DsxF and DsxM proteins respectively were introduced into *Drosophila melanogaster*, and their effect on somatic sexual development in the ensuing transgenic flies recorded. The *Anastrepha* DsxF and DsxM proteins allowed partial female and male sexual determination respectively in *Drosophila*. However, the extent of their influence was not the same in the different sexually dimorphic regions of the adult fly.

## Results

### The DsxF and DsxM proteins of *Anastrepha* supply partial female and male sexual determination function, respectively, in transgenic *Drosophila* flies

To analyse the effect of the *Anastrepha dsx* gene in *Drosophila*, the GAL4-UAS system was used. *AodsxF*-cDNA and *AodsxM*-cDNA was linked to UAS sequences. As expected, none of the *Aodsx* transgenic *Drosophila* lines expressed the corresponding transgene in the absence of GAL4. If any basal expression existed, this would be irrelevant since XX and XY flies with one or two doses of each transgene are normal, fertile females and males respectively.

A set of different GAL4 driver lines was used to express the transgenic AoDsx proteins Tub-GAL4, Arm-GAL4 and C68a-GAL4 [20]. The first two drives expression ubiquitously whilst the latter one is specific for imaginal discs. It was found that, independent of the GAL4 driver used, the expression of either AoDsxF or AoDsxM proteins was lethal to the transgenic flies when these were raised at 25°C (both males and females died at the embryonic and early larval stages). This lethality was not suppressed in those transgenic flies lacking the endogenous *dsx* function, i.e., mutants for *dsx* (data not shown). A similar result was reported when the proteins of *Drosophila* [21] or *Ceratitis capitata* [12] DsxM protein were expressed in *Drosophila* transgenic flies. The effectiveness of the GAL4-UAS system depends on temperature: lower temperature reduces the effectiveness of GAL4 so that the expression of the UAS-transgene is reduced. Therefore, the transgenic flies were raised at either 18 or 22°C, although only some of the transgenic flies expressing either the AoDsxF or the AoDsxM proteins survived to adulthood (see Tables 1 and 2). These were processed as explained in Materials and Methods, so that the effect of the transgenic proteins on *Drosophila* somatic sexual development could be studied. To this end, the following sexually dimorphic regions of the fly were monitored: the foreleg basitarsus, the 5th, 6th and 7th tergites, and the external terminalia. In all cases, the control refers to either XX or XY *dsx* mutant flies and experiment refers to their XX sisters or XY brothers mutant for *dsx* but expressing the *Anastrepha* Dsx proteins.

**Table 1:** Frequency and size of external structures in the terminalia of *D. melanogaster* flies expressing the *Anastrepha* DsxF female protein.

Cross	Genotype	Female genital structures		Male genital structures				AP
		T8	VP	GA	LP	CL	PA	
			Frequency (x±SEM)	Frequency (x±SEM)	Frequency (x±SEM)	Frequency (x±SEM)		
I (18°C)	*yw /w; FAo#2 / CyO; dsx^1^ / dsx^1^* (28)	1.0	1.0 (3.8±0.6)	0.96 (4.6±0.8)	0.42 (17.1±5.6)	0.25 (28.0±7.3)	reduced	intersexual
	*yw /w; FAo#2 / C68a-GAL4; dsx^1^ / dsx^1^* (25)	1.0	1.0 (17.2±1.0)	0.44 (4.5±0.5)	0.44 (11.3±2.9)	0.40 (17.5±2.5)	reduced	intersexual
	*yw /Y; FAo#2 / CyO; dsx^1^ / dsx^1^* (24)	1.0	1.0 (2.2±0.8)	0.83 (7.7±1.5)	0.75 (32.3±3.6)	0.75 (35.5±2.3)	reduced	intersexual
	*yw /Y; FAo#2 / C68a-GAL4; dsx^1^ / dsx^1^* (27)	1.0	1.0 (18.3±0.9)	1.0 (6.0±0.6)	1.0 (24.3±2.7)	1.0 (30.4±2.1)	reduced	intersexual
II (22°C)	*ywFAo#10 /w; CyO/+; dsx^1^ / dsx^1^* (43)	1.0	1.0 (5.7±0.4)	1.0 (7.6±0.8)	0.86 (39.1±2.6)	0.86 (37.5±2.5)	reduced	intersexual
	*ywFAo#10 /w; arm-GAL4/+; dsx^1^ / dsx^1^* (25)	1.0	1.0 (23.4±1.5)	0.36 (6.3±0.7)	0.6 (28.4±2.9)	0.6 (24.0±3.2)	reduced	intersexual
III (18°C)	*yw /Y; FAo#2 / C68a-GAL4; FAo#1 / MKRS,Sb* (19)	1.0	0.9 (4.8±0.8)	1.0 (9.7±0.3)	1.0 (37.3±1.7)	1.0 (33.6±0.9)	reduced	intersexual
	*yw /Y; FAo#2 / C68a-GAL4; FAo#1 / dsx^1^* (22)	1.0	1.0 (6.5±0.9)	1.0 (5.7±0.5)	1.0 (31.0±1.9)	1.0 (24.8±1.6)	reduced	intersexual

Symbols: T8, 8th tergite; VP, vaginal plates; GA, genital arch; LP, lateral plates; CL, clasper; PA, penis apparatus comprising the penis proper and hypandrium; AP, anal plates. The number in parenthesis following the genotype indicates the number of analysed flies. Frequency refers to the presence of the corresponding structure. The size was calculated by counting the number bristles in the different structures. Crosses in Materials and Methods.

**Table 2:** Frequency and size of external structures in the terminalia of *D. melanogaster* flies expressing the *Anastrepha* DsxM male protein.

Cross	Genotype	Female genital structure		Male genital structure				AP
		T8	VP	GA	LP	CL	PA	
			Frequency (x±SEM)	Frequency (x±SEM)	Frequency (x±SEM)	Frequency (x±SEM)		
IV (18°C)	*yw /w; MAo#10 / arm-GAL4; dsx^1^ / dsx^1^* (23)	1.0	0.78 (2.6±0.4)	1.0 (10.9±0.4)	1.0 (42.5±1.0)	1.0 (39.8±1.8)	reduced	intersexual
	*yw /Y; MAo#10 /arm-GAL4; dsx^1^ / dsx^1^* (17)	1.0	0.23 (1.7±0.4)	1.0 (10.1±0.4)	1.0 (39.7±1.2)	1,0 (40.8±1.2)	reduced	intersexual
V (18°C)	*yw /w; MAo#10/C68a-GAL4; MAo#4/MKRS,Sb* (25)	0.88	1,0 (21.4±1.1)	0.0	0.0	0.0	reduced	intersexual
	*yw /w; MAo#10/C68a-GAL4; MAo#4 / dsx^1^* (36)	0.69	1,0 (10.4±0.7)	0.80 (6.6±1.4)	0.11 (17.5±3.5)	0,11 (20.0±5.0)	reduced	intersexual

Symbols: T8, 8th tergite; VP, vaginal plates; GA, genital arch; LP, lateral plates; CL, clasper; PA, penis apparatus comprising the penis proper and hypandrium; AP, anal plates. The number in parenthesis following the genotype indicates the number of analysed flies. Frequency refers to the presence of the corresponding structure. The size was calculated by counting the number bristles in the different structures. Crosses in Materials and Methods.

**Figure 1:**
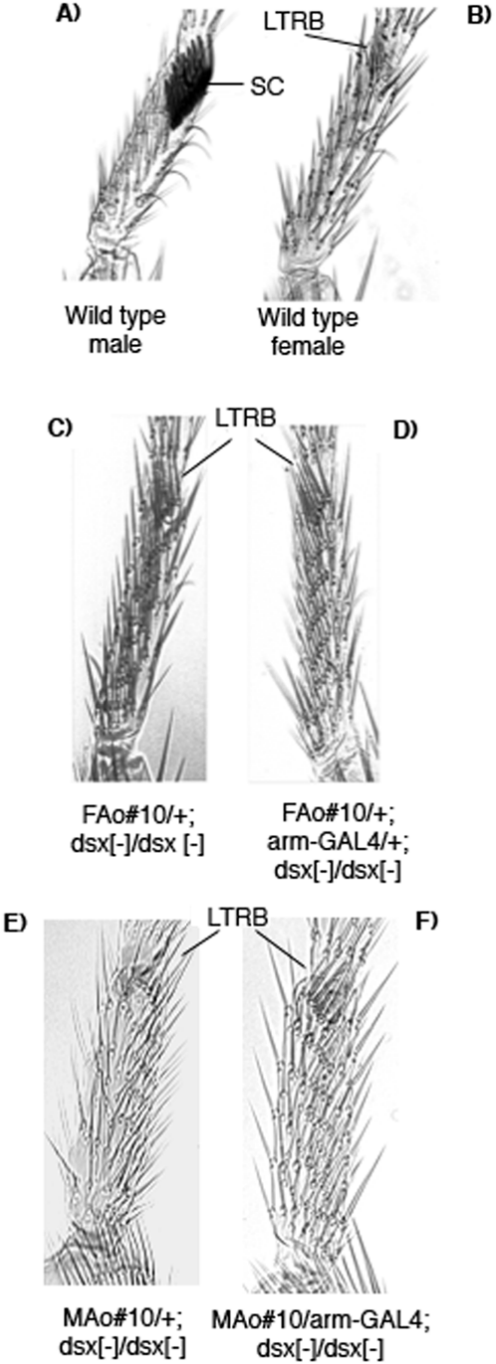
Morphological analysis of the foreleg basitarsus of *Anastrepha dsx* transgenic *Drosophila* flies. (A) Wild type male. (B) Wild type female. (C) XX flies of genotype *ywFAo#10/w; CyO/+; dsx^1^/dsx^1^*. (D) XX flies of genotype *ywFAo#10/+;arm-GAL4/+; dsx^1^/dsx^1^*. These latter two genotypes correspond to sister flies from cross II (see Materials and Methods). (E) XY flies of genotype *yw/Y; MAo#10/CyO; dsx^1^/dsx^1^*. (F) XY flies of genotype *yw/Y; MAo#10/arm-GAL4; dsx^1^/dsx^1^*. These latter two genotypes correspond to brother flies from cross IV (see Materials and Methods). Symbols: SC, sex comb; LTRB, last transversal row of bristles.

In wild type flies, the foreleg basitarsus contains several transversal rows, the last one forming the sex comb structure in males (SC in Figure 1A); this is absent in females. The sex comb is composed of dark, thick bristles, and is rotated to lie parallel to the proximal-distal leg axis. In XX and XY flies mutant for *dsx*, no sex comb is formed and the last transversal row of bristles (LTRB in Fig. 1B,C,D,E,F) is partially rotated and formed by bristles that are not as dark and thick as the sex comb bristles. In the present work, the phenotype was the same when *dsx* mutant XX and XY flies expressed either the *MAo* or the *FAo* transgene. See examples of control *FAo#10/+; dsx^1^/dsx^1^* (Figure 1C) and its experimental sister *FAo#10/+;arm-GAL4/+; dsx^1^/dsx^1^* (Figure 1D), non-expressing and expressing the transgene respectively, and control *MAo#10/+; dsx^1^/dsx^1^* (Figure 1E) and its experimental brother *MAo#10/+;arm-GAL4/+; dsx^1^/dsx^1^* (Figure 1F), non-expressing and expressing the transgene respectively. This suggests that neither the DsxF nor the DsxM proteins of *Anastrepha* have an effect on the sexual development of the *Drosophila* foreleg basitarsus. In the case of C68a-GAL4 driver (specific for imaginal discs) some survivors presented forelegs with morphological abnormalities (including partial duplications), whose occurrence is characteristic of cell death during development (data not shown).

**Figure 2:**
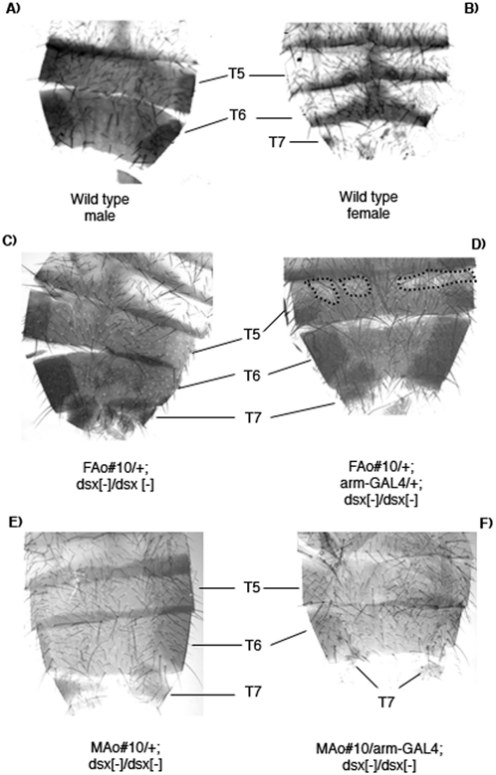
Morphological analysis of the abdomen of *Anastrepha dsx* transgenic *Drosophila* flies. (A) Wild type male. (B) Wild type female. (C) XX flies of genotype *ywFAo#10/w; CyO/+; dsx^1^/dsx^1^*. (D) XX flies of genotype *ywFAo#10/+;arm-GAL4/+; dsx^1^/dsx^1^*. These latter two genotypes correspond to sister flies from cross II (see Materials and Methods). (E) XY flies of genotype *yw/Y; MAo#10/CyO; dsx^1^/dsx^1^*. (F) XY flies of genotype *yw/Y; MAo#10/arm-GAL4; dsx^1^/dsx^1^*. These latter two genotypes correspond to brother flies from cross IV (see Materials and Methods). Symbols: T4–T7, tergite 4–tergite 7.

The 5th and 6th tergites of wild type males are fully pigmented (Fig. 2A) whereas in females only the posterior area is pigmented (Fig. 2B). In XX and XY flies mutant for *dsx*, the 5th tergite is intersexual and characterised by the presence of pigmented spots in the anterior area. The 6th tergite shows the male-like dark pigmentation. In our *dsx^1^* mutant stock, the 5th tergite showed a more male-like colouring, with only small, non-pigmented spots in the most anterior and lateral regions (see *FAo#10/+; dsx^1^/dsx^1^* in Fig. 2C). Usually, In transgenic flies mutant for *dsx* and expressing either the *MAo* or *FAo* transgenes, the 5th and 6th tergites showed a slight sexual transformation towards male or female respectively (see *FAo#10/+;arm-GAL4/+; dsx^1^/dsx^1^* in Fig. 2D, which shows a larger non-pigmented area in the most anterior region that is marked by a dotted line). This indicates that these transgenes had a small effect on the development of 5th and 6th tergites of *dsx* mutant flies.

**Figure 3:**
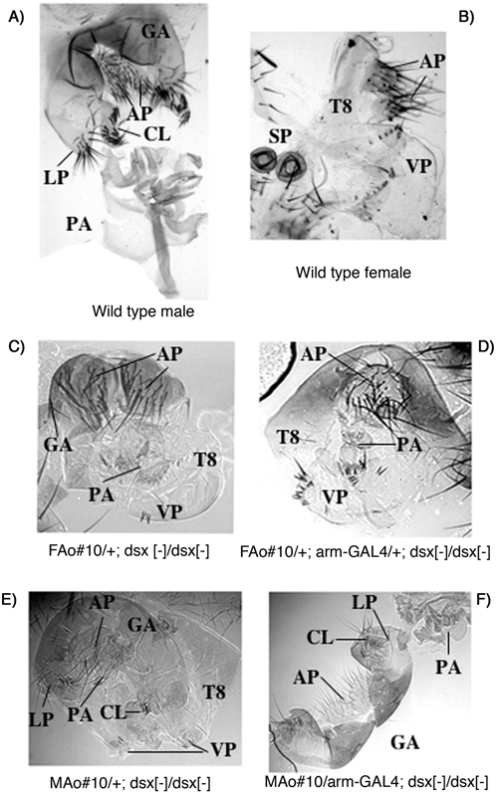
Morphological analysis of the external adult terminalia of *Anastrepha dsx* transgenic *Drosophila* flies. (A) Wild type male. (B) Wild type female. (C) XX flies of genotype *FAo#10/+; dsx^1^/dsx^1^*. (D) XX flies of genotype *FAo#10/+;arm-GAL4/+; dsx^1^/dsx^1^*. (E) XY flies of genotype *dsx^1^/dsx^1^*. (F) XY flies of genotype *arm-GAL4/MAo#10; dsx^1^/dsx^1^*. The C and D genotypes correspond to sister flies from cross II, whereas genotype E is the offspring of cross I and genotype F the offspring of cross IV (see Materials and Methods). Symbols: T8, 8th tergite; VP, vaginal plates; SP, spermathecae; GA, genital arch; LP, lateral plates; CL, clasper; PA, penis apparatus comprising the penis proper and hypandrium; AP, anal plates.

The 7th tergite is present in wild type females and in *dsx* mutant XX and XY flies, although in these it is smaller. In the present work, this tergite could be still smaller in *dsx* mutant flies expressing the *MAo* transgene (Figure 3F), whereas in *dsx* mutant flies expressing the *FAo* transgene a more female-like development could be observed (Figure 3D).

The most conspicuous sexually dimorphic region of the fly is the external terminalia (Fig. 3A,B), which are derived from the genital disc (reviewed in [22], [23]). This is composed of two genital primordia plus the anal primordium. In both sexes, only two of these primordia develop to form the adult terminalia. The anal primordium develops in both sexes but, depending on the genetic sex, will form either male or female analia. However, only one of the genital primordia develops in each sex, forming either the male or the female adult genitalia. This depends on the genetic sex of the fly, i.e., the production of either female DsxF or male DsxM protein. In loss-of-function *dsx* mutant flies - whether XX or XY - both genital primordia develop giving rise to variable intersexual terminalia with incomplete male and female genital structures and intersexual analia [24], [25], [26] (see Fig. 3C,E).

Table 1 shows the results corresponding to the effect of the *FAo* transgene on the development of the external terminalia of *dsx* mutant XX and XY flies. The loss-of-function *dsx^1^* mutation used in this study caused a slightly different degree of intersexuality in XX and XY flies (Fig. 3C, E). This can be appreciated by comparing rows 1 and 3 of Table 1; male genital structures were more common and larger in XY than in XX flies mutant for *dsx^1^*. For this reason, the effect of the transgene was compared between XX sister flies, or between XY brother flies, expressing (or not) the transgene. The expression of the *Anastrepha* DsxF protein in both *dsx* mutant XX and XY flies caused a female transformation of the intersexual terminalia, though this was incomplete. In the case of the flies expressing the *FAo#2* transgene and reared at 18°C (cross I, Table 1), the expression of this transgene determined an increase of the size of the female vaginal plates and a reduction in the size of the male genital arch, lateral plate and clasper structures. This transformation towards female sex is more evident in the case of the *FAo#10* transgene in *dsx* mutant XX flies reared at 22°C (cross II, Table 1) (Fig. 3D). Both the frequency and the size of the male genital structures decreased. Their XY brothers did not survive, probably because the *FAo#10* transgene is located on the X chromosome (and therefore dose compensated), and thus males express larger amounts of transgenic DsxF protein – which is lethal, as explained at the beginning of this section. It may also well be that the GAL4 driver line *arm-GAL4* is more active than *C68a-GAL4*. The female sexual transformation was also manifested by the presence of female spermathecae (absent in *dsx* mutant flies) in some transgenic flies expressing either the *FAo#2* or *FAo#10* transgenes, and by a large reduction in the size of the penis apparatus.

Table 2 shows the effect of the *MAo* transgene on the development of the external terminalia of *dsx* mutant XX and XY flies. The expression of the *Anastrepha* DsxM protein in both *dsx* mutant XX and XY flies caused male transformation of the intersexual terminalia, though this was incomplete. As a control, the intersex phenotype of *dsx^1^/dsx^1^* flies of cross I (Table 1) was used. The size of the female vaginal plates decreased whereas the size of the male genital arch, lateral plates and claspers increased. In some cases, neither T8 nor the vaginal plates were present (Fig. 3F).

Collectively, the morphological analyses suggest that the *Anastrepha* DsxF and DsxM proteins cause feminisation and masculinisation respectively of *dsx* intersexual *Drosophila* flies, though this transformation was incomplete and the extent of their influence varied in the different sexually dimorphic regions of the adult fly, suggesting that the partial sex-determination function of the *Anastrepha* Dsx proteins in *Drosophila* reflect a reduced capacity of the *Anastrepha* Dsx proteins to completely control the *Drosophila* sexual cyto-differentiation genes in the different sexually dimorphic regions.

### The *Anastrepha* DsxF and DsxM proteins counteract the function of *Drosophila* DsxM and DsxF respectively

An indistinguishable intersexual phenotype is attained when both DsxF and DsxM are either absent or simultaneously present whenever they are in similar amounts. If one of the Dsx proteins is in greater quantity, it determines the sexual development that the zygote will follow [27], [28]. This is so because DsxF and DsxM behave as antagonistic transcriptional factors in the regulation of their common target genes (reviewed in [4]). We were interested in studying the capacity of the *Anastrepha* DsxF protein to compete with the endogenous *Drosophila* DsxM protein. To examine this, XY brothers expressing two *FAo* transgenes (*FAo#2* and *FAo#1*) and carrying either two doses (*dsx^+^ / dsx^+^*) or one dose (*dsx^1^ / dsx^+^*) of the endogenous *Drosophila dsx* gene were produced (cross III, Table 1). These express the same amount of *Anastrepha* DsxF protein and either the normal amount or half the amount of *Drosophila* DsxM protein respectively. The XY transgenic flies with two doses of endogenous *dsx* (row 7, Table 1) showed some female genital structures such as T8 tergite and vaginal plates as well as a reduction of the penis apparatus and intersexual analia. Their brothers with one dose of endogenous *dsx* (row 8, Table 1) showed a significant increment in the size of the vaginal plates and a significant reduction in the size of the male genital arch, lateral plate and clasper structures, in addition to a large reduction in the size of the penis apparatus. In addition, the analia showed a greater degree of intersexuality. Hence, it appears that the transgenic *Anastrepha* DsxF protein can partially counteract the effect of the *Drosophila* DsxM protein, this effect being more intense the less of the latter protein there is.

The capability of the *Anastrepha* DsxM protein to compete with the endogenous *Drosophila* DsxF protein was analysed in sister XX flies expressing two *MAo* transgenes (*MAo#10* and *MAo#4*) and carrying either two doses (*dsx^+^ / dsx^+^*) or one dose (*dsx^1^ / dsx^+^*) of the endogenous *Drosophila dsx* gene (cross V, Table 2). The XX flies with two doses of endogenous *dsx* showed some minor degree of intersexuality, manifested in their intersexual analia and the presence of a reduced penis apparatus always enclosed by the vaginal plates. Their brothers with one dose of endogenous *dsx* showed an increment in their intersexuality, observable by a reduction in the size of the vaginal plates along with an increase in the size of the penis apparatus, an increase in the intersexuality of the analia, and the presence of a male genital arch and lateral plate and clasper structures. Therefore, it appears that the transgenic *Anastrepha* DsxM protein is able to partially counteract the effect of the *Drosophila* DsxF protein, this effect being more intense the less of the latter protein there is.

### Effect of the DsxF and DsxM proteins of *Anastrepha* on the regulation of *Drosophila yolk protein* genes

This is the new paragraph: The *yolk protein (yp)* genes of *Drosophila* are co-ordinately transcribed in the fat body of adult females under the control of *dsx*. DsxF and DsxM act as activator and repressor, respectively, by binding to the same regulatory sequences (reviewed in [29]). These genes are also expressed in the follicle cells of the ovary, although it appears that they are no longer under the control of *dsx* but are regulated by tissue specific factors present in those cells [30]. In loss-of-function *dsx* mutant XX flies lacking both DsxF and DsxM proteins, basal transcription of the *yp* genes in the fat body (gonads are not developed) has been reported [31], though in our *dsx^1^* stock such remnant expression was not observed.

**Figure 4:**
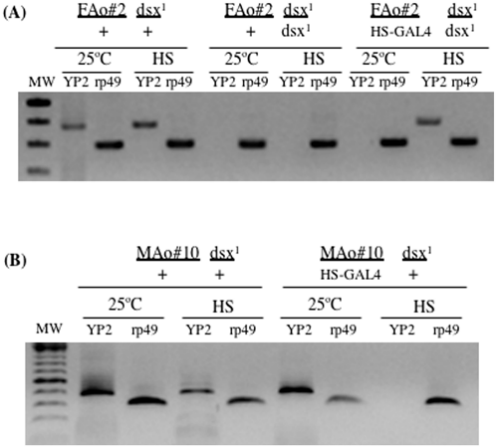
Expression of *Drosophila yolk protein 2* gene *Anastrepha dsx* transgenic *Drosophila* flies. The genotypes in (A) and (B) correspond to the offspring of crosses VI and VII respectively (see Materials and Methods). *yp2* and *rp49* stand for the *yolk protein 2* and *ribosomal protein 49* genes respectively. ‘25°C’ indicates that the flies were maintained at this temperature after eclosion whereas HS indicates that they were subject to two 3 h heat shock pulses (37°C) per day for two consecutive days with recovery at 25°C between pulses. PCR amplification of total RNA extracts (without cDNA) yielded no amplification product, indicating that the RNA sample was devoid of DNA.

The effect of *Anastrepha* DsxF protein on the regulation of *Drosophila yp* genes was studied my monitoring the expression of *yp2* in transgenic *Drosophila* XX flies mutant for *dsx* and expressing the *Anastrepha* DsxF protein. The inducible HS-GAL4 driver was used to express the *FAo#2* transgene. XX flies of genotype *FAo#2/+; dsx^1^/+* (control females), *FAo#2/+; dsx^1^/dsx^1^* (intersexual flies) and *FAo#2/HS-GAL4; dsx^1^/dsx^1^* (experimental flies) were produced at 25°C (cross VI in Materials and Methods). After the eclosion of the adults, each class of females was divided into two populations; one was maintained at 25°C and the other subjected to heat-shock pulses to induce the expression of the transgene. All three classes of females received heat shock treatment at the same time (see legend to Fig. 4A). Total RNA was extracted and used in RT-PCR to determine the expression of *yp2* and the expression of *rp49* (which codes for the constitutive ribosomal protein 49) [32] (used as a control; for details see Materials and Methods). The results are presented in Figure 4. As expected, the control females expressed the *yp2* gene whereas intersexual flies did not, whether kept at 25°C or subject to heat shock. Neither did the experimental females express the *yp2* gene when maintained at 25°C, although they did express it after heat shock. The three classes of females expressed the control *rp49* gene when kept at 25°C and after heat shock. These results indicate that the *Anastrepha* DsxF protein behaves as an activator of the *Drosophila yp* genes, just like DsxF of *Drosophila*.

To determine whether the *Anastrepha* DsxM protein acts as an inhibitor of the *yp* genes, as does that of *Drosophila* itself, normal, XX, fertile females of genotype *MAo#10/+; dsx^1^/+* (control females) and *MAo#10/HS-GAL4; dsx^1^/+* (experimental females) were raised at 25°C (cross VII in Materials and Methods). The same experimental plan described above was followed. Both control and experimental females produced the *Drosophila* endogenous DsxF protein, while the experimental females also produced the transgenic *Anastrepha* DsxM protein when subjected to the heat shock. As expected, the control females expressed the *yp2* gene whether maintained at 25°C or when subjected to heat shock. The experimental females also expressed the *yp2* gene when maintained at 25°C. These females, however, did not express this gene after heat shock, i.e., when the transgenic *Anastrepha* DsxM protein is produced (Fig. 4B). These results show that this protein counteracts the activation of the *yp* genes by the endogenous *Drosophila* DsxF protein.

## Discussion

This investigation provided the following major results. 1) The *Anastrepha* DsxF and DsxM proteins cause feminisation and masculinisation respectively of *dsx* intersexual *Drosophila* flies, though this transformation is incomplete and their influence varies between the different sexually dimorphic structures. 2) The *Anastrepha* DsxF and DsxM proteins behave as activator and repressor respectively, of the *Drosophila yolk protein* genes, as do the DsxF and DsxM proteins of *Drosophila* itself. 3). The *Anastrepha* DsxF and DsxM proteins are able to counteract the function of *Drosophila* DsxM and DsxF respectively, just as these latter proteins behave towards one another. Collectively, these results indicate that the *Anastrepha* DsxF and DsxM proteins show conserved female and male sex-determination function respectively in *Drosophila*. Nevertheless, it appears that they cannot fully substitute the *Drosophila* Dsx proteins.

This incomplete function might be partly due to the insufficient amount of *Anastrepha* Dsx proteins produced in *Drosophila* transgenic flies, since the presence of two doses (rather than one) of *AodsxF* or *AodsxM* transgenes enhanced the corresponding modification towards female or male. It should be remembered that the GAL4/UAS system, the effectiveness of which depends on temperature, was used to express the *Anastrepha dsx* transgene, and that the flies had to be raised at 18 or 22°C to allow the transgenic *Drosophila* flies to reach adulthood and sexual transformation to be monitored. More complete sexual transformation might be obtained by allowing the production of greater amounts of *Anastrepha* Dsx proteins, but this could not be tested since the transgenic *Drosophila* died at higher temperatures.

There is, however, a clear-cut result; namely, the different developmental response of sexually dimorphic regions to the function of *Anastrepha* Dsx proteins. This cannot be explained by substantially different amounts of these proteins being present, since the GAL-4 driver is constitutively expressed in these structures. Rather, the present results suggest that the partial sex-determination function of the *Anastrepha* Dsx proteins in *Drosophila* reflect a reduced capacity of the *Anastrepha* Dsx proteins to completely control the *Drosophila* sexual cytodifferentiation genes in the different sexually dimorphic regions.

The development of sexually dimorphic structures not only depends on gene *dsx* but on an integrated signal involving the corresponding Dsx protein – either male or female - and the appropriate homeotic protein that determines segmental specificity. Thus, the sexual phenotype of the prothoracic leg basitarsus (i.e., either formation of the sex comb in males or its absence in females) requires, besides *dsx*, additional inputs from the homeotic gene *Sex comb reduced (Scr)* (which specifies prothoracic identity) and from the *Distal-less* gene (which specifies proximal-distal identity) [21]. The sexual phenotype of the abdominal tergites requires, besides *dsx*, inputs from the homeotic gene *Abdominal-B* [33], [34]. Finally, besides *dsx*, the sexual development of the genital disc requires inputs from the homeotic genes *Abdominal-B* [35], [36], [37] and *caudal* [38], [39]. Therefore, the dissimilar effect of the *Anastrepha* Dsx proteins on the development of different *Drosophila* sexually dimorphic regions may be a consequence of the accumulation of divergence between these species resulting in the formation of different co-adapted complexes between the Dsx proteins and the target genes.

In the above context, the following results are important. Firstly, the expression of *Musca domestica* DsxM protein in *Drosophila* XX flies does not affect normal female development except for variable male-like pigmentation in the 5th and 6th tergites in some flies [7]. In contrast, the expression of *Ceratitis capitata* DsxM protein in *Drosophila* females induces partial masculinisation [12], as does *Anastrepha* DsxM protein (this work). *Ceratitis* and *Anastrepha* belong to the family Tephritidae, whereas *Musca* belongs to Muscidae. The molecular evolution of gene *dsx* in insects shows that the Dsx proteins of the tephritids are more closely related to those of *Drosophila* than to those of *Musca* Dsx [14]. Together, these results suggest that the evolutionary divergence among Dsx proteins is greater between *Musca* and *Drosophila* than between *Drosophila* and the tephritids.

Secondly, the DsxF protein needs to interact with the Intersex protein in order to perform its function [40], [41], [42]. The *Musca* [7] and the *Anastrepha* (this work) DsxF protein can induce the synthesis of *Drosophila yolk protein* genes, indicating that they can interact with the *Drosophila* Intersex protein. It is thus suggested that the dissimilar function of the tephritid and *Musca* Dsx proteins in *Drosophila* might be due to different evolutionary changes in these proteins and/or the other regulatory proteins involved in the integrated signal dictating the developmental route the sexual dimorphic structures will follow. It should also be remembered that the *D. melanogaster* flies simultaneously expressing DsxF and DsxM proteins show both male and female genital structures and intersexual analia [24], [25], [26]. In interspecific hybrids expressing the *Drosophila teissieri* DsxF protein and the *Drosophila melanogaster* DsxM (*teissieri–melanogaster* hybrids), however, the external genitalia might be defined as more male-like than intersexual. Indeed, they have an almost completely normal set of male genital structures. Nonetheless, the analia remain intersexual [43]. To explain the male-like phenotype of the genitalia of these hybrids, the latter authors speculated that during the evolution of the *D. melanogaster* and *D. teissieri* species, genetic changes occurred in regulatory genes such as *dsx* and/or *Abd-B*, and/or in the genes controlled by these regulators, all of which are responsible for the development of the terminalia. These species-specific variations might be responsible for the morphological changes observed in the terminalia of these species. When the genotypes of the two species are put together within a hybrid cell, divergent co-adapted gene complexes confront one another. This might result in the formation of hybrid patterns different from those of either parental species; i.e., in the production of morphological diversity [43].

Finally, recent molecular data supports the formation of different co-adapted complexes between the Abd-B and Dsx proteins and its target genes. It has been found that *Abd-B* and *dsx* act in concert upon the *cis*-regulatory element (CRE) of the gene *bric-à-brac (bab)* to control the sexually dimorphic development of the 5th and 6th tergites in *Drosophila melanogaster*. In females, Abd-B and DsxF activate *bab*, whereas in males DsxM represses it, thus allowing for male-specific pigmentation. This genetic control evolved through changes within the CRE element of gene *bab* [34]. For a discussion of CREs changes in morphological evolution see Carroll [44].

In conclusion, it is proposed that the different sensitivity of the different sexually dimorphic regions of *Drosophila* to the *Anastrepha* Dsx proteins reflects the accumulation of different evolutionary changes not only in the *Dsx* proteins of these two species but also in their genes specifying segmental identity. As a result, the integrated genetic input determining the sexual development of each of these dimorphic regions is affected in different way.

## Materials and Methods

### Flies and crosses

Flies were cultured on standard food. For the description of the mutant alleles or GAL4 constructs see Lindsley and Zimm [45] and FlyBase. Flies used for the analysis of adult forelegs, abdomens and external terminalia were kept in a mixture of ethanol∶glycerol (3∶1) for several days. They were then macerated in 10% KOH at 60°C for 15 min, thoroughly washed with water and mounted in Faure's solution for inspection under a compound microscope. *FAo* and *Mao* stand for *UAS::AodsxF*-cDNA and *UAS::AodsxM*-cDNA, respectively. The crosses were:

1. Females *yw; FAo#2; dsx^1^ / MKRS,Sb* and males *w/Y; C68a-GAL4 / CyO; dsx^1^ / MKRS,Sb*
2. Females *ywFAo#10; dsx^1^ / MKRS,Sb* and males *w/Y; arm-GAL4 / CyO; dsx^1^ / TM3,Sb*
3. Females *yw; FAo#2; FAo#1* and males *w/Y; C68a-GAL4 dsx^1^ / MKRS,Sb*
4. Females *yw; MAo#10; dsx^1^ / MKRS,Sb* and males *w/Y; arm-GAL4 / CyO dsx^1^ / TM3·,Sb*
5. Females *yw; MAo#10; MAo#4* and males *w/Y; C68a-GAL4 dsx^1^ / MKRS,Sb*
6. Females *yw; FAo#2; dsx^1^ / MKRS,Sb* and males *w/Y; HS-GAL4 / CyO; dsx^1^ / MKRS,Sb*
7. Females *yw; MAo#10; dsx^1^ / MKRS,Sb* and males *w/Y; HS-GAL4 / CyO; dsx^1^ / MKRS,Sb*

### Construction of *UAS::AodsxF*-cDNA and *UAS::AodsxM*-cDNA transgenes

For the construction of the *UAS::AodsxF*-cDNA and *UAS::AodsxM*-cDNA transgenes, a fragment of 1568 bp, or 1579 bp, comprising the whole ORF of *Anastrepha obliqua dsxF*, or *dsxM*, was amplified by RT-PCR using a common primer at the 5′UTR (5′GTGAGTCAGGGTTTAGCTC3′) and a female-specific primer (5′GTCATTGTTCCGCAAACATGG3′) or a male-specific primer (5′CAGTGAGTCAGGGCTTTAGC3′) at the corresponding 3′UTR. The amplicon was cloned in the TOPO-TA cloning vector (Invitrogen). The cDNA fragments were then digested with Eco *RI* and cloned in *pUAST* vector [20]. The microinjections for generating the *FAo* and *MAo* transgenic *Drosophila melanogaster* lines were performed by Genetic Services (Sudbury, MA, USA). Standard genetic crosses determined the chromosomal location of the transgenes. To ascertain that each transgenic line was carrying the correct transgene, PCR on genomic DNA was used to amplify the whole transgene and the amplicons were cloned and sequenced.

### Molecular analyses

Total RNA extracts from frozen adults were prepared using the Ultraspec-II RNA isolation kit (Biotecx) following the manufacturer's instructions. Five micrograms of total RNA from each sample were reversed transcribed with Superscript II (Invitrogen) following the manufacturer's instructions. Reverse transcription reactions were performed with an oligo-dT. Two percent of the synthesised cDNA was amplified by PCR. The amplicons were analysed by electrophoresis in agarose gels. The primers used in the PCR for the analysis of the *yp2* expression in the *Drosophila* transgenic flies were (5′GTCGTTGAGGCCACCATGC3′) and (5′GGAGTGGTTCGCTCGCATG3′), which amplify a fragment of 368 bp. As a control, the expression of gene *rp49* [32] was monitored by PCR using the same cDNA sample used for the analysis of *yp2*. The PCR primers used for *rp49* were (5′ATCCGCCACCAGTCGGATC3′) and (5′TGGCGCGCTCGACAATCTC3′), which amplify a fragment of 286 bp. Ten percent of the cDNA was used for PCR in a total volume of 50 µl. The PCR conditions were 95°C, 2 minutes, followed by 45 cycles of 95°C for 45 s, 59°C for 45 s, and 72°C for 1 min, plus an extension step at 72°C for 1 min. Fifteen microlitres of the *yp2* PCR reaction volume and 8 µl of the *rp49* PCR reaction volume were loaded onto gels for electrophoresis.
